# Nutrition, mental health and violence: from pregnancy to postpartum Cohort of women attending primary care units in Southern Brazil - ECCAGE study

**DOI:** 10.1186/1471-244X-10-66

**Published:** 2010-08-31

**Authors:** Maria A Nunes, Cleusa P Ferri, Patricia Manzolli, Rafael M Soares, Michele Drehmer, Caroline Buss, Andressa Giacomello, Juliana F Hoffmann, Silvia Ozcariz, Cristiane Melere, Carlo N Manenti, Suzi Camey, Bruce B Duncan, Maria I Schmidt

**Affiliations:** 1 Graduate Program in Epidemiology, Universidade Federal do Rio Grande do Sul, Rua Ramiro Barcelos, 2400 - 2° andar - 90035-003 - Porto Alegre/RS, Brazil; 2 Section of Epidemiology, Institute of Psychiatry, HSPR, King's College, 16 De Crespigny Park London SE5 8AF, UK; 3 Statistics Department, Universidade Federal do Rio Grande do Sul, Av. Bento Gonçalves, 9500 - Prédio 43-111 - Agronomia, 91509-900 Porto Alegre/RS, Brazil

## Abstract

**Background:**

Woman's nutritional status, before and during pregnancy, is a strong determinant of health outcomes in the mother and newborn. Gestational weight gain and postpartum weight retention increases risk of overweight or obesity in the future and they depend on the pregestational nutritional status and on food consumption and eating behavior during pregnancy. Eating behavior during pregnancy may be the cause or consequence of mood changes during pregnancy, especially depression, which increases likelihood of postpartum depression. In Brazil, a study carried out in the immediate postpartum period found that one in three women experienced some type of violence during pregnancy. Violence and depression are strongly associated and both exposures during pregnancy are associated with increased maternal stress and subsequent harm to the infant. The main objectives of this study are: to identify food intake and eating behaviors patterns; to estimate the prevalence of common mental disorders and the experience of violence during and after pregnancy; and to estimate the association between these exposures and infant's health and development.

**Methods/Design:**

This is a cohort study of 780 pregnant women receiving care in 18 primary care units in two cities in Southern Brazil. Pregnant women were first evaluated between the 16^th ^and 36^th ^week of pregnancy at a prenatal visit. Follow-up included immediate postpartum assessment and around the fifth month postpartum. Information was obtained on sociodemographic characteristics, living circumstances, food intake, eating behaviors, mental health and exposure to violence, and on infant's development and anthropometrics measurements.

**Discussion:**

This project will bring relevant information for a better understanding of the relationship between exposures during pregnancy and how they might affect child development, which can be useful for a better planning of health actions aiming to enhance available resources in primary health care.

## Background

A woman's nutritional status, before and during pregnancy, is a strong determinant of health outcomes in the mother and newborn, and can affect the infant's structure, physiology and metabolism[1,2]. Both maternal mental health and exposure to violence during pregnancy, which are closely related, may also be risk factors for adverse neonatal [3] and infant's health outcomes [4]. There is a need to better understand the role of these exposures during and after pregnancy and the potential pathways linking them to the newborn and infants health.

### Diet

The Family Budget Research of 2002-2003[5] showed that Brazilian households' diet is poor in healthy foods which contributed to an increase in incidence of obesity and other chronic diseases. It also showed an increase in consumption of carbohydrate and fat and reduction in consumption of grains and beans which are typical foods in Brazilian diet and that the average amount of fruits and vegetables available for consumption in households corresponded to approximately 1/3 of recommended values. It has been shown that socioeconomic factors, especially income and schooling are important determinants of the Brazilian urban population's dietary patterns [6]. Studies evaluating food intake of women during pregnancy are rare in Brazil. A cohort of Brazilian pregnant women (mean age of 25 years) attending the public health network showed mean energetic consumption of 2,889 kcal during pregnancy and 2,081 kcal in the postpartum period. Except for coffee, wine, beer and alcohol, which had an increase in postpartum diet, all the other energy values, macronutrients, micronutrients, fatty acids and cholesterol had a statistically significant fall between pregnancy and postpartum (9-month follow-up). In contrast to eating patterns during pregnancy, characterized by a diet high in industrialized or fast preparation foods, consumption of more traditional foods in the Brazilian diet such as rice and beans increased in the postpartum period. This is a positive finding as a dietary pattern based on consumption of rice and beans seems to be protective and not associated with weight excess [7]. Two Brazilian studies measured food intake in pregnant adolescent showing an important nutritional unbalance with excessive consumption of lipids, and adolescent who were in the lowest quartile of consumption did not reach the minimum recommended consumption of energy and nutrients[8,9].

There are a high number of studies evaluating dietary patterns during pregnancy in developed countries. In Denmark two dietary patterns during pregnancy were identified. The first is characterized by red meat and products with high percentage of fat, and the second dietary pattern is characterized by intake of fruits, vegetables, birds, and fish. The first pattern was associated with low fetal development [10]. We have not found any study from Brazil looking specifically on dietary patterns during pregnancy.

### Gestational weight gain and postpartum retention

Weight gain in pregnancy, both insufficient and excessive, is associated with duration of pregnancy, type of delivery, newborn's weight, and weight retention in the postpartum period; therefore, it is an indicator of maternal-fetal health. Postpartum weight retention is determined by pregestational nutritional status and by excessive weight gain, characterized by inappropriate food consumption and eating behavior during pregnancy. It is associated with increased risk of overweight or obesity up to 15 years later[11]. With the current epidemic of obesity and evidence relating maternal nutrition with occurrence of chronic disease in adulthood[12], high incidence of excessive weight gain should be considered as a public health problem[11-13].

Studies of pregnant women receiving care in primary health services showed a 37.8% incidence of insufficient gestational weight gain and 29.2% of excessive weight gain [14,15]. Maternal weight gain below expected values is associated with low-weight newborns and longer hospital stay. Abrams et al. found that pregnant women with insufficient weight gain during the third trimester were 2.46 times (95%CI: 1.53-3.92) more likely to have spontaneous premature delivery in relation to pregnant women that gained weight within recommended values. Excessive weight gain, on the other hand, is associated with higher incidence of macrosomia, cesarean delivery and child obesity[16]. Another study showed odds ratio of 1.40 (95%CI: 1.22-1.59) for cesarean delivery in pregnant women with excessive weight gain [16-18].

### Food intake

Although there are many methods to quantify food intake, obtaining reliable and valid data in epidemiological nutritional studies is a hard task because there is no gold standard [19]. So far there is no consensus as to the best form of evaluating an individual's food intake. Dietary recall and food frequency questionnaire have been applied to evaluate the diet of pregnant women [19-21]. The Food Frequency Questionnaire (FFQ) is widely used. It measures long periods of time, has fast application and easy analysis when compared with dietary records and recall. These advantages represent low cost, which is particularly important in epidemiological studies [8,22]. The FFQ which was previously validated for pregnant women was used in this study [23].

### Mental health

The mean age of first pregnancy in Brazil is 16.8 years old. This poses women to an early development of inappropriate eating behaviors (food restrictions, use of diuretics and laxatives, self-induced vomiting, and episodes of excessive food intake) and eating disorders (anorexia nervosa and bulimia nervosa) which is more common among women in young age groups and occurs in about 1% of pregnant women[24]. Episodes of excessive food intake may occur during the gestational period and are usually followed by excessive weight gain. Some studies found prevalence of these episodes in primigravid women of 25-44% [25,26]. Inappropriate eating behaviors are commonly followed by other psychiatric symptoms, such as anxiety and depression [27,28]. Occurrence of inappropriate eating behaviors and eating disorders may contribute to maternal and fetal complications, such as intrauterine growth retardation, prematurity, low birth weight, hyperemesis gravidarum, gestational diabetes, preeclampsia/hypertension, high frequency of cesarean delivery, and low Apgar scores[29-32].

Among psychiatric disorders, the most common are depressive and anxiety disorders, known as common mental disorders (CMD). They contribute to 1/3 of work absence due to diseases and to 1/5 of all primary care visits, which shows how much these disorders are disabling and a public health concern[33]. Depression is the most prevalent women mental health disorder and a very important health problem overall; suicide, for instance, was the second cause of death in 1990 among women aged 15-44 years old after tuberculosis [34]. Its occurrence during pregnancy poses further risks to women [35] as it is associated with obstetric complications, such as premature labor, preeclampsia, bleeding, and premature rupture of membranes[36] and postpartum depression[28,37].

It is estimated that 25-35% of pregnant women have depressive symptoms and that 20% of them may meet the diagnostic criteria for major depression [38,39]. Studies conducted in Brazil showed that the prevalence of any psychiatric disorder during pregnancy is 27.6% [40] and of depressive disorder is 19.1% [28,37]. Despite its high prevalence, depression during pregnancy is undetected and associated with less prenatal care and poor nutrition [41]. There is also a strong association between depression and consumption of alcoholic beverages and smoking during pregnancy [42-44], further increasing the risk to which mother and baby are exposed.

Maternal depression in the pre- and postnatal periods predicts an impaired infant development, risk of early interruption of breastfeeding[45] and high rates of recurrent diarrhea in newborns[4], in addition to affecting the intelectual and psychological development of the child[46].

### Violence

It is estimated that one out of three women is victim of some type of violence in childhood, adolescence, or adult life [47]. Although controversial, pregnancy has been considered a period of increased risk for violence[48]. Gazmararian et al., in a systematic review, found that prevalence of violence during pregnancy is 1-20%[49]. In Brazil a study conducted in the immediate postpartum period found that 33.8% of puerperal women had suffered some type of violence during pregnancy[50]. Violence can be a trigger for depression and anxiety symptoms; and although reverse causality is an issue, the prevalence of depression during pregnancy has been shown to be four times higher in pregnant women exposed to violence compared to women not exposed[51].

The consequences of exposure to violence during pregnancy may have a direct influence on the woman's health, leading to risk behaviors, such as consumption of alcoholic beverages and drugs, and delayed onset of prenatal care [52]. Violence can also compromise pregnancy outcomes, increasing risk of premature labor and presenting a two-fold risk of low birth weight [3,53,54]. Other studies showed association between violence during pregnancy and a poor diet, risk of anemia and lower maternal weight gain[55,56], in addition to increased risk of developing depressive disorder[57].

### Conclusion

Pre- and post-natal are periods of increased vulnerability to the occurrence of mental disorders, such as depression and anxiety; behavior disturbances including poor diet habits and alcohol and tobacco intake, affecting mother and infant well-being[58]. There is a lack of Brazilian cohort studies among pregnant women using primary health care in general and especially studies on maternal nutrition and mental health. There is a need to disentangle the interrelationship between these different exposures during pregnancy and how they might affect mother and infant health to inform clear guidance on nutritional choices and prevention of both violence and mental disorders during and after pregnancy.

### Objectives

This project main objectives are to identify food intake and eating behavior patterns, estimate common mental disorders and experience of violence during and after pregnancy and to estimate the association of these exposures with maternal and infant's health and development. More specifically we will test the following hypothesis:

a) Violence and common mental disorders are highly prevalent during the pre- and the post-natal period; b) Violence and common mental disorders during pregnancy are associated with obstetric complications, such as premature labor, preeclampsia, bleeding, and low birth weight; c) Violence and common mental disorders during pregnancy is associated with mental disorder in the postpartum period; d) Maternal depression in the pre- and post-natal periods are associated with infant development impairment. e) Early interruption of breastfeeding mediates the association described in (d); f) Deficient nutrition during and after pregnancy partially explains the association in (b).

## Method/Design

### Population

In Brazil primary health services are the entry door to the Brazilian Unified Health System (SUS) (SMS 2006-2008). They provide basic health guidance, home visits, and referral for more complex examinations, surgeries or medical specialties. Primary health services prioritize promotion and prevention of health, and provide pregnant women with free follow-up during the prenatal period by a multidisciplinary team (Municipal Department of Health, SMS).

The ECAGE Project (Study of Food Intake and Eating Behavior of Pregnant Women) was conducted in two cities (Bento Gonçalves (city 1) and Porto Alegre (city 2)) in the southernmost state in Brazil, It has a population of 10,582,840 inhabitants, child mortality rate of 13.20/1000 liveborns (FEE RS, 2006), life expectancy at birth of 72.05 years (FEE RS, 2000), illiteracy rate of 6.65% (FEE RS, 2000), unemployment rate of 6.4% [59] (IBGE/Brazilian Household Sampling Survey - PNAD 2005), and GDP per capita of R$ 15,812.55 (FEE RS, 2007). The State public health system provides 2.02 physicians for each 1,000 inhabitants (Department of Health - CGRH-SUS/SIRH, 2005) and has 913 health units distributed across the State. Approximately 24,267,069 visits are performed in the outpatient health system, reaching a mean of 2.24 visits per inhabitant (Source: Department of Health/SE/Datasus 2005 - Outpatient Information System of SUS (SIA/SUS).

### Study design

This is a cohort study of pregnant women attending 18 primary care units in the State of Rio Grande do Sul, Brazil. Participant's first assessment was conducted between the 16^th ^and 36^th ^week of pregnancy at a prenatal care visit. Follow-up included immediate and at fourth- fifth month postpartum assessment.

### Baseline

Enrollment was conducted at the waiting room prior to the prenatal consultation from June 2006 to April 2007. 780 pregnant women were consecutively invited to participate, of whom 68 (8.7%) refused to participate, totaling a sample of 712 women at baseline. Inclusion criteria were having prenatal care in one of the selected locations and gestational age between 16 and 36 weeks. The baseline interview was performed after medical prenatal care visit by a trained interviewer at a single contact with the participants.

### Baseline measures

#### Sociodemographic

Data on participants and their partners regarding age, schooling, socioeconomic status, housing, and life style (tobacco and alcohol consumption) were obtained.

#### Obstetric history

number of pregnancies, number of children, planned pregnancy, tobacco and alcohol consumption during pregnancy, and preexisting clinical conditions, such as hypertension and diabetes. Pregnant women also had their weight and height measured.

#### Pre-natal history

Data were collected from pregnant women regarding all prenatal visits that included weight, blood pressure, uterine height, gestational age, duration of pregnancy, ultrasounds, and events during pregnancy based on medical records in basic health care units or in hospitals.

#### Mental Health

*Common mental disorders *were evaluated using the Primary Care Evaluation of Mental Disorders (PRIME-MD)[60], which has been used to screen, evaluate and diagnose mental disorders in primary health care, translated and validated to Brazilian Portuguese[61]. The instrument comprehends mood disorder, anxiety, somatoform disorders, eating disorders, and likely alcohol dependence. *Eating Behaviors*: Eating Disorder Examination - Questionnaire (EDE-Q)[62,63], validated into Portuguese[64]. This instrument provides screening of eating disorder symptoms, derived from a semi structured diagnostic interview called EDE, widely used in studies on eating disorders.

#### Experience of violence

The questionnaire to evaluate violence was developed based on the instrument Abuse Assessment Screen [65,66], which investigates psychological (humiliations and verbal offenses), physical (with or without gunfire) and sexual (being forced to perform any type of sex) violence throughout life and during current pregnancy. Data were collected on the life cycle in which the event occurred, perpetrator, and whether there was search of help for each type of violence. This part of the questionnaire was self-reported to increase response quality and rate; interviewers had no access to the information. Main exposure to violence will be defined as violence suffered during pregnancy and categorized as: never, psychological only, physical only, psychological and physical.

#### Diet

Data on food intake were collected through an 88-item Food Frequency Questionnaire, developed by Sichieri and Everhart [67] and validated for this population [23].

### Immediate Postpartum

The pregnant women were contacted by telephone using the information on the likely date of delivery. Information on women without telephone contact was obtained from the Information System of Liveborns (SINASC) which is a national system of information recording data on details of births in hospitals. 711 interviews were conducted (only one participant lost to follow-up). A review of medical records at the basic health unit was also conducted. 708 prenatal recordes were examined (only 4 were not found).

### Immediate Postpartum measures

Details of birth included delivery date and location, type of delivery, hospital stay, and obstetric events. Information on the infant included gender, weight and length, maternal breastfeeding, and if there were any events. APGAR score will be defined as below 7 at 5 minutes [68]. Birth weight was obtained in grams and low-birth-weight will be defined as 2500 g or less as suggested by the World Health Organization [69]. Small for gestational age will be defined as a birth weight below the 10th percentile [70]. The cut-off point for premature delivery will be defined as 37 weeks of gestation age [69]. Gestational age was calculated by routine ultrasonography conducted during prenatal care. When gestational age is above 20 weeks on the date of ultrasonography, we will use the mean value between gestational age obtained by ultrasonography and that obtained by the date of the last menstrual period. When ultrasonography was not available, gestational age will be calculated according to the date of the last menstrual period.

### Interview at 4-5 months postpartum

The interviews were scheduled by telephone and carried out at the basic health units where mother and child were having their post-natal care. Exceptionally the interview happened at the participant's house using a semi structured questionnaire, with the same baseline instruments, adding questions on the infant's health and development. This study stage was performed on women living in Porto Alegre and Bento Gonçalves (city 2; n = 401. city 1; n = 61), totaling a sample of 462 women. Three women had three twins. Twenty-five women were lost to follow-up (9 refused to participate and 16 were not found) totaling a sample of 434 women at the follow-up). After three unsuccessful attempts of telephone contact, participants received a domiciliary visit

### Measures at 5-6 months postpartum (mother)

Eating behaviors, mental disorders, experience of violence, tobacco and alcohol consumption were measured as in the baseline (see baseline section above for details). Measures on breastfeeding practices (frequency, duration, reasons for interruption, etc); social and financial support to help with infant care and weight and height measures were obtained.

### Measures at 5-6 months postpartum (infant)

Infant's development at 5-6 months: introduction of foods, hospitalizations and clinical diseases, vaccine schedule, and anthropometric measurements (weight, length and head circumference) were evaluated. Information from the mother on infant's neuropsychomotor development (sustaining the head, following objects with their eyes, turning in bed without help, listening when called for, playing with their hands, and recognizing presence of people) was also collected.

Anthropometric infant measurements: growth measurements (weight-for-age and height-for-age) will be standardized to generate z-scores using the 2006 WHO reference population [71]. This outcome will be used as a continuous measure (z-scores) and will also be dichotomized to define those undernourished by using the cut-off of -2.

### Data entry

The software Teleform^® ^(Cardiff, Vista, California) was used to create the questionnaire.

Data were input on a weekly basis. The questionnaires were scanned and then converted into images in the SPSS 13.0 using the Teleform^®^. Checking for errors in the database was performed upon data entry.

### Quality Control

Quality control of the interview occurred in 10% of the sample, selected at random both in the baseline and in the follow-up through telephone contact. A reduced version of the original instrument was applied, comprised of identification variables, five sociodemographic items, three from the FFQ, five from the EDE-Q, and two items of the PRIME-MD. Three questions about the infant were added to the questionnaire of follow-up quality control.

### Sample size

Several calculations were performed using the tool STATCALC of Epi-Info to set sample size for ECCAGE in the baseline. The largest sample calculated to estimate a prevalence of inadequate eating behavior was 10%, with 95% confidence interval and absolute error of 2.3%, resulting in 654 pregnant women. There was a 20% increment to compensate for possible losses and/or refusals, resulting in a total of 785 participants. This sample size (5% alpha and 80% power) allowed for estimating RR higher than 1.81 for a ratio between non-exposed and exposed of approximately 3:1. By the end of the baseline study the lack of financial resources reduced follow-up for just above half of the original sample. This sample size (n = 459) (5% alpha and 80% power) allowed for estimating RR higher than 2.05 for a ratio between non-exposed and exposed of approximately 3:1.

### Ethical aspects

The participants and/or parents/guardians (when the pregnant woman was under 14 years of age) signed a consent term at a private site. In case the woman was illiterate, the interviewer read the term. This project was approved by the Research Ethics Committee of Universidade Federal do Rio Grande do Sul and by similar committees governing research in the health care services under study. Written informed consent was obtained from all participants.

### Statistical analysis

Statistical analysis will be carried out using SPSS version 16.0 package. R version 2.4.1 and AMOS version 7.0. Descriptive analysis of data will be performed by means and standard deviation for quantitative variables and frequency and percentage for categorical variables. Poisson regression will be used to evaluate associations between possible risk factors and outcomes, with robust variance for binary outcomes and multinomial logistic regression for polytomous outcomes. The models will be adjusted for potential confounders. For example, for the association between exposure during pregnancy and neonatal outcome, potential confounders include: maternal age, education, family income, gestational age, gestational weight gain, alcohol and tobacco.

Analysis of principal components (varimax rotation) and cluster analysis will be used to evaluate eating patterns. A model on the potential causal relationship between different exposures and outcome will be built and tested using Structural Equation Modeling [72-74].

## Discussion

In Brazil over the past decades there have been remarkable advances in basic maternal and child care, with remarkable improvement in health indicators, such as access to prenatal care, incentive to breastfeeding, vaccine coverage, and most relevantly reduced mortality during the first year of life. However, there is still room for more advances in maternal and child outcomes. By combining different areas, such as maternal nutrition, mental health and violence against pregnant women, this project brings relevant information for a better understanding of the relationship between exposures during pregnancy and maternal health and child development. We believe that findings of this study have the potential to influence both clinical practice and public health prevention efforts.

## Competing interests

The authors declare that they have no competing interests.

## Authors' contributions

MAAN had full access to all of the data in the study and takes responsibility for the integrity of the data and the accuracy of the data analysis. Study concept and design: MAAN, CF, MIS Acquisition of data: PM, CM, RS, MD, CB, AG, JH, SO, CM. Analysis and interpretation of data of the study: AG, RS, CB, PM, MD, JH, SO, CM. Drafting of the manuscript: MAAN, CF, PM, RS, MD, CB, JH, SO. Critical revision of the manuscript for important intellectual content: MAAN, CF, MD, PM. Responsible for the statistical and analytic aspects of the study: SC. All authors read and approved the final manuscript.

## Pre-publication history

The pre-publication history for this paper can be accessed here:

http://www.biomedcentral.com/1471-244X/10/66/prepub
